# Unconditional or Conditional Logistic Regression Model for Age-Matched Case–Control Data?

**DOI:** 10.3389/fpubh.2018.00057

**Published:** 2018-03-02

**Authors:** Chia-Ling Kuo, Yinghui Duan, James Grady

**Affiliations:** ^1^Connecticut Institute for Clinical and Translational Science, University of Connecticut Health Center, Farmington, CT, United States; ^2^Department of Community Medicine and Health Care, University of Connecticut Health Center, Farmington, CT, United States

**Keywords:** frequency matching, individual matching, sparse data problem, loose matching, precision in estimates and tests, width of 95% confidence interval

## Abstract

Matching on demographic variables is commonly used in case–control studies to adjust for confounding at the design stage. There is a presumption that matched data need to be analyzed by matched methods. Conditional logistic regression has become a standard for matched case–control data to tackle the sparse data problem. The sparse data problem, however, may not be a concern for loose-matching data when the matching between cases and controls is not unique, and one case can be matched to other controls without substantially changing the association. Data matched on a few demographic variables are clearly loose-matching data, and we hypothesize that unconditional logistic regression is a proper method to perform. To address the hypothesis, we compare unconditional and conditional logistic regression models by precision in estimates and hypothesis testing using simulated matched case–control data. Our results support our hypothesis; however, the unconditional model is not as robust as the conditional model to the matching distortion that the matching process not only makes cases and controls similar for matching variables but also for the exposure status. When the study design involves other complex features or the computational burden is high, matching in loose-matching data can be ignored for negligible loss in testing and estimation if the distributions of matching variables are not extremely different between cases and controls.

## Introduction

Matching is commonly used in case–control studies to adjust for confounding at the design stage. It ensures that adjustment is possible when there is no sufficient overlap in confounding variables between cases and a random set of controls. Earlier literature often describes the advantages of matching in case–control studies as adjusting for confounding and improving the study efficiency (1–4). Other reasons to match include control of unmeasured confounders and ensuring statistical power to perform subgroup analysis and to test for interactions (5). The study efficiency is improved if a smaller sample size is needed for the same precision or a narrower confidence interval is obtained using the same sample size. Previous studies have compared efficiency of matched and unmatched studies (3, 6–8). The comparison is complex in that the efficiency is affected not only by matching but also by other factors that are difficult to specify in advance (9). In summary, matching is efficient if the matching variables are true confounders and if only a moderate number of controls must be dropped because they cannot be matched to a case (9).

Age, sex, and race are common confounders as suggested by descriptive epidemiology (5). The distributions of these variables may substantially differ between cases and controls, and a random sample of controls may lead to spurious associations due to confounders. Matching is a method to tackle the problem, and there are two types of matching: frequency matching and individual matching. In frequency matching, controls are selected such that cases and controls have similar distributions of matching variables. In individual matching, matching is performed for cases individually assuming the majority in the population are controls. Given a particular case, the matched controls can be selected following exact matching, e.g., matching on sex, or interval matching, e.g., matching on age by within 3 years of the case’s age (age ± 3 years). Usually, the case–control matching ratio is fixed and preselected. While an increasing number of controls would increase precision in estimates and tests, the marginal improvement is negligible from a ratio beyond 4, except when the effect of exposure is large (5).

Matching may incur the sparse data problem that requires the use of matched methods. When the sample size is not sufficiently large relative to the number of strata where each matching set forms a stratum statistically, the sparse data problem arises and causes the estimate to bias away from the true value (10). Conditional logistic regression was developed as a remedy for the sparse data bias and has become a standard for analyzing matched case–control data (11). We argue that there are circumstances when the number of strata is large compared to the sample size but the sparse data problem does not exist. In a case–control study that investigates the association between cancer risk and exposure to asbestos, age is known to be a true confounder and assume cases and controls are matched on age by age ± 3 years. While the exposure frequency of asbestos significantly differs between young and old subjects at the two ends, the difference is minimal between subjects who are only a few years apart. Subjects with similar ages thus can be grouped into a stratum without introducing bias to the association. With a decreasing number of strata, the sparse data problem is largely relieved, and unmatched methods become appropriate in theory.

Our research is motivated by one of the two misconceptions discussed by Pearce that if matching has been performed, then a “matched analysis” is required (12). Pearce conducted a simple experiment that mimicked a paired matched case–control study where each case was matched to a control from the same age group (young or old). Both matched and unmatched analyses yielded similar results. He thus concluded that pair matched analysis is not required unless cases and controls are genuinely matched, e.g., using siblings as controls or matching on many factors simultaneously. In this article, we extensively study the misconception by simulation where one case is matched to controls with a similar age, and the association and the effect of confounding vary. We hypothesize that matching on demographic variables typically generates “loose-matching” data, which can be appropriately analyzed by an unmatched method. In loose-matching data, one case can be matched to other controls without substantially changing the association. The rematching may occur obeying or beyond the matching criteria, which implies that matching itself is not statistically efficient. It is not straightforward, and we have no attempts to quantify loose matching. Instead, we simulate matched case–control data that mimic real data and meet the loose-matching definition. Our goal is to show that unmatched methods are appropriate for matched case–control data that are essentially loose-matching data.

## Materials and Methods

### Statistical Methods

Denote by *Y* the case–control status, where *y* = 1 if a case and *y* = 0 if a control. Denote by *****X******_m_* = {*****X******_m_*_1_, *****X******_m_*_2_} a vector of matching variables where variables in *****X******_m_*_1_ are exactly matched and variables in *****X******_m_*_2_ are interval matched. Denote by *X_e_*, an exposure to associate with the case–control status, and *****X******_o_*, a vector of unmatched variables to include in the model. Denote by *S* the id of matching set. *s* = *i* for subjects in the *i*th matching set for *i* = 1, 2, …, *n*. In the unconditional logistic regression, the model assuming no interaction is given by

(1) $$\text{logit}(\pi )=\beta_{0}+\beta_{e}x_{e}+\beta_{m}^{T}x_{m}+\beta_{o}^{T}x_{o},$$

where π is the probability of developing the disease, and β’s are the associated regression coefficients. Correspondingly, the conditional logistic regression model is given by

(2) $$\text{logit}(\pi )=\beta_{0i}+\beta_{e}x_{e}+\beta_{m_{2}}^{T}x_{m_{2}}+\beta_{o}^{T}x_{o},$$

where β_0_*_i_* denotes the contribution to the logit of all terms constant within the *i*th matching set and other parameters are as those defined in the unconditional model in Eq. 1 (11). The interval matching variables need to be controlled in the conditional model because the matching process makes cases and controls similar not only for the matching variables but also for the exposure status (12, 13). In each model, the two-sided *P* value against the null hypothesis, H_0_: β*_e_* = 0, is recorded as well as the β*_e_* estimate, here denoted by ${\hat{\beta }}_{e}$.

### Simulations

We simulated matched case–control data to test for the association between a binary exposure and the case–control status of a disease. We assumed that the exposure was the only predictor and age was the only confounder. One case was matched to *k* controls, and the number of cases was *n*_1_. Throughout this article, “case” is referred to as the outcome status of case in case–control studies.

Denote by *p_e_* the exposure frequency. Given the exposure status, the distribution of age (*x_a_*) was approximated by a normal distribution, *N*(μ_0_, σ^2^) for unexposed subjects and *N*(μ_1_, σ^2^) for exposed subjects. Given the exposure status and age, the disease risk was modeled by

(3) $$\text{logit}(\pi )=\beta_{0}+\beta_{e}x_{e}+\beta_{a}x_{a},$$

where *x_e_* was 1 if exposed and 0 if unexposed. β*_e_* and β*_a_* were specified *via* the odds ratios of the exposure and age. β_0_ was chosen such that the disease prevalence was controlled at *K*. Based on the model in Eq. 3, the probability of developing the disease given the exposure status and age was

(4) $$\pi (x_{e},x_{a})=Pr(Y=1|x_{e},x_{a})=\frac{exp(\beta_{0}+\beta_{e}x_{e}+\beta_{a}x_{a})}{1+exp(\beta_{0}+\beta_{e}x_{e}+\beta_{a}x_{a})}.$$

A forward simulation was conducted to simulate the data of cases. We first simulated exposed and unexposed subjects followed by their ages and then case–control statuses based on the disease probability in Eq. 4. The simulated age was truncated to its smallest following integer due to the perception of age. The simulation continued until collecting sufficient cases to power the study.

We assumed that the population size is unlimited, and every case can be matched a control. To facilitate the case–control matching, we simulated the exposure statuses and ages of matched controls from the distribution of exposure status and age for controls aged in the matching range. Let the age of a case be *u* and the matched controls with an age within {*u*_1_, *u*_2_} = {*u − d, u* + *d*}, where both *u* and *d* are integers. The exposure status and age of every matched control were jointly simulated from

(5) $$\text{Pr}(x_{e},x_{a}|y=0,x_{a}\in ℛ)=\frac{\text{Pr}(x_{e},x_{a},y=0)}{\sum_{i=0}^{1}\sum_{j=u_{1}}^{u_{2}}P\text{r}(x_{e}=i,x_{a}=j,y=0)},$$

where *x_e_* = 0, 1, and *x_a_* = *u − d, u − d* + 1,…, *u* + *d −* 1, *u* + *d*. In the denominator,

(6) $$\begin{array}{l} \text{Pr}(x_{e}=i,x_{a}=j,y=0)=\text{Pr}(x_{e}=i)\\ \;\left[\Phi (j+1|\mu_{i},\sigma^{2})-\Phi (j|\mu_{i},\sigma^{2})\right]\times \left[1-\pi (x_{e}=i,x_{a}=j)\right], \end{array}$$

where Φ(•) is the cumulative density function of a normal distribution governed by a mean and a SD.

**Figure 1:**
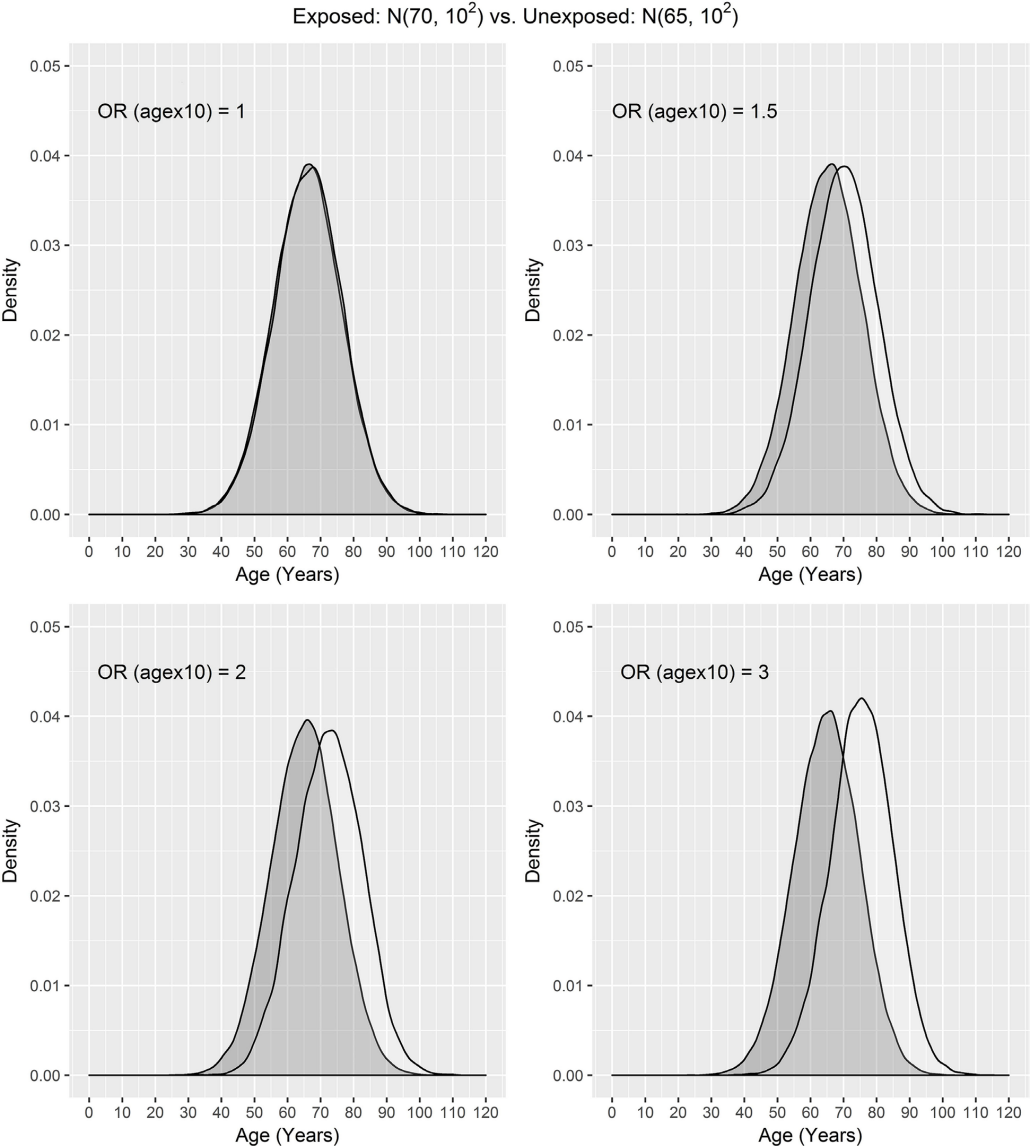
Age distributions of cases (white) and controls (grey) in the population, where the age distributions of exposed and unexposed subjects are *N*(70, 10^2^) and *N*(65, 10^2^), respectively, and OR (agex10) denotes odds ratio associated with a 10-year increase in age.

**Figure 2:**
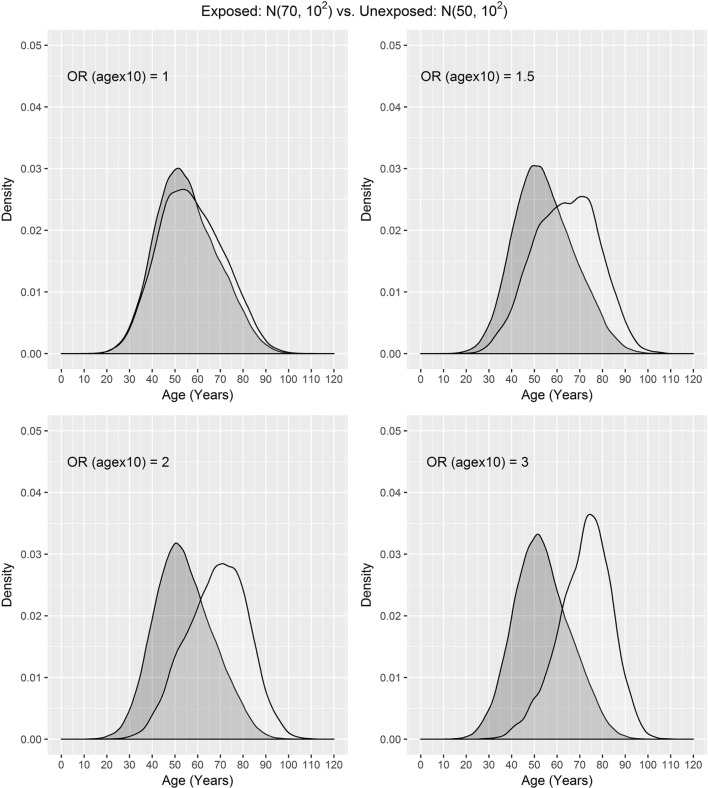
Age distributions of cases (white) and controls (grey) in the population where the age distributions of exposed and unexposed subjects are *N*(70, 10^2^) and *N*(50, 10^2^) and OR (agex10) denotes odds ratio associated with a 10-year increase in age.

In our settings, we considered a disease with the prevalence of 10%. We assumed that the exposure frequency was 30%, the age distribution of unexposed subjects was *N*(μ_0_, σ^2^), and the age distribution of exposed subjects was *N*(μ_1_, σ^2^), where μ_0_ = 50, 60, 65 and μ_1_ = 70. The odds ratio associated with the exposure was set to 1.5, and the odds ratio associated with a 10-year increase in age was 1, 1.5, 2, or 3. One case was matched to 1, 2, 3, or 4 controls by age ± *d*, where *d* = 0, 1, 2, and 3. The age distributions of cases and controls are presented using a population sample that contains 10,000 cases (Figures 1 and 2) for the settings of μ_0_ = 65, 50. The age distributions for μ_0_ = 60 are in between the distributions for μ_0_ = 65, 50 and are not presented here for saving space.

The significance level was set to 5% to test against H_0_: β*_e_* = 0. 1,000 data sets were used when the alternative hypothesis (*H*_1_: β*_e_* ≠ *0*) was true. 10, 000 data sets were simulated instead when the null hypothesis was true. Each data set contained a number of matching sets (one case and one control). Each matching set contributed data to age, exposure, and outcome. The number of matching sets was chosen for a power around 80% across simulation settings, i.e., 400, 500, and 900 when age distributions of exposed and unexposed subjects were 5, 10, and 20 years apart. Specifically, the sample size was roughly determined by simulations. More simulation replicates were required to provide sufficient accuracy for type I errors around 5%. The unconditional and conditional models were fitted to each data set and were compared across data sets by type I error and power for testing and by bias and width of 95% confidence interval for estimation.

## Results

In simulations, we manipulated the confounding effect of age by the odds ratio associated with a 10-year increase in age and by the mean difference in age between exposed and unexposed subjects. The results are consistent regardless of case–control matching ratio. Therefore, we only present the results of 1:1 matching.

### Hypothesis Testing

For the hypothesis test on β*_e_*, we compare the unconditional and conditional models by type I error under the null hypothesis, H_0_: β*_e_* = 0 and by power under the alternative hypothesis, *H*_1_: β*_e_* ≠ 0. In Table 1, we present the type I error results. A type I error is considered reasonable if falling in the 95% confidence interval for the nominal level of 5%: 0.0457, 0.0543. The power simulation results are presented in Table 2. The two models are considered equally powerful if the absolute power difference is smaller than 5%.

**Table 1:** Type I errors of unconditional and conditional logistic regression models.

	Age distribution of unexposed and exposed subjects (in years)					
	*N*(65, 10^2^) vs. *N(*70, 10^2^)		*N*(60, 10^2^) vs. *N(*70, 10^2^)		*N*(50, 10^2^) vs. *N(*70, 10^2^)	
*d*	Unconditional	Conditional	Unconditional	Conditional	Unconditional	Conditional
**Odds ratio associated with a 10-year increase in age = 1**						
0	0.048	0.048	0.046	0.046	**0.040**	0.053
1	0.052	0.051	0.047	0.048	**0.038**	0.052
2	0.049	0.049	0.049	0.050	**0.036**	0.049
3	0.051	0.051	0.050	0.050	**0.034**	0.048
**Odds ratio associated with a 10-year increase in age = 1.5**						
0	0.050	0.049	0.050	0.050	**0.041**	0.051
1	0.048	0.046	**0.044**	**0.044**	**0.038**	0.049
2	0.050	0.049	0.051	0.051	**0.040**	0.052
3	0.052	0.050	0.052	0.051	**0.040**	0.049
**Odds ratio associated with a 10-year increase in age = 2**						
0	0.051	0.050	0.051	0.051	**0.038**	0.047
1	0.048	0.049	0.051	0.050	**0.039**	0.050
2	0.053	0.053	0.046	0.046	**0.037**	0.047
3	0.052	0.049	0.047	0.048	**0.039**	0.048
**Odds ratio associated with a 10-year increase in age = 3**						
0	0.049	0.048	**0.045**	**0.046**	**0.039**	0.050
1	0.048	0.047	0.051	0.052	**0.038**	0.050
2	0.047	**0.045**	0.050	0.050	**0.041**	0.053
3	0.052	0.052	0.050	0.050	0.046	0.053

*Cases and controls were matched by age ± d. The odds ratio associated with the exposure was 1 under the null hypothesis, H_0_: β_e_ = 0. Numbers of matching sets were 400, 500, and 900 in the three scenarios of age distributions. Type I errors out of the 95% confidence interval for the nominal level of 5% were highlighted in bold, 0.0457, 0.0543*.

**Table 2:** Power of unconditional and conditional logistic regression models.

	Age distribution of unexposed and exposed subjects (in years)					
	*N*(65, 10^2^) vs. *N(*70, 10^2^)		*N*(60, 10^2^) vs. *N(*70, 10^2^)		*N*(50, 10^2^) vs. *N(*70, 10^2^)	
*d*	Unconditional	Conditional	Unconditional	Conditional	Unconditional	Conditional
**Odds ratio associated with a 10-year increase in age = 1**						
0	0.73	0.73	0.78	0.77	0.78	0.80
1	0.77	0.76	0.76	0.77	0.78	0.81
2	0.73	0.72	0.76	0.75	0.78	0.81
3	0.73	0.73	0.77	0.78	0.78	0.80
**Odds ratio associated with a 10-year increase in age = 1.5**						
0	0.76	0.76	0.80	0.80	0.82	0.84
1	0.75	0.74	0.81	0.82	0.79	0.83
2	0.80	0.80	0.81	0.80	0.81	0.83
3	0.78	0.78	0.82	0.82	0.81	0.84
**Odds ratio associated with a 10-year increase in age = 2**						
0	0.80	0.79	0.80	0.80	0.76	0.78
1	0.79	0.79	0.83	0.82	0.81	0.83
2	0.79	0.79	0.82	0.80	0.78	0.82
3	0.78	0.77	0.80	0.80	0.75	0.76
**Odds ratio associated with a 10-year increase in age = 3**						
0	0.76	0.76	0.83	0.83	0.77	0.80
1	0.80	0.80	0.85	0.85	0.76	0.78
2	0.79	0.78	0.82	0.82	0.75	0.79
3	0.81	0.78	0.85	0.83	0.71	0.74

*Cases and controls were matched by age ± d. The odds ratio associated with the exposure was 1.5 under the alternative hypothesis, H_0_: β_e_ ≠ 0. Numbers of matching sets were 400, 500, and 900 in the three scenarios of age distributions. Power simulation results that gave a difference between models 5% or greater were highlighted in bold*.

When the mean age difference is 5, i.e., age distribution *N*(65, 10^2^) for unexposed subjects and *N*(70, 10^2^) for exposed subjects, the type I error consistently falls in the acceptable range (left panel in Table 1). The only out-of-the-range type I error most likely occurring by chance is from the conditional model when the age matching range is age ± 2. The unconditional models consistently give similar power with an absolute difference smaller than 5% (left panel in Table 2).

When the mean age difference is 10, i.e., age distribution *N*(60, 10^2^) for unexposed subjects and *N*(70, 10^2^) for exposed subjects, the results are consistent with those when the mean difference is 5. Both models give reasonable type I errors. Except for a couple of scenarios, they produce type I errors below the range (middle panel in Table 1). Both models are equally powerful when the alternative hypothesis is true (middle panel in Table 2).

When the mean age difference is 20, i.e., age distribution *N*(50, 10^2^) for unexposed subjects and *N*(70, 10^2^) for exposed subjects, the conditional model consistently maintains a reasonable type I error, while the unconditional model gives a type I error below the range (right panel in Table 1). Both models perform similarly in terms of power (right panel in Table 2). The unconditional model, however, is consistently less powerful than the conditional model. When the odds ratio associated with a 10-year increase in age is 3, the power is decreasing with a wider matching range of age. This is not observed until the confounding effect is large.

### Estimation

We compare the unconditional and conditional models in the estimation of β*_e_* by bias or percent of bias and width of 95% confidence interval. We let the odds ratio associated with the exposure be 1 under the null hypothesis and 1.5 under the alternative hypothesis, equivalent to β*_e_* = ln 1.5 = 0.0405 under the alternative hypothesis.

Over the simulation replicates, we collect the β*_e_* estimates to calculate the percent of bias (% bias) when the alternative hypothesis is true by

(7) $$\%bias=\frac{1}{n_{r}}\overset{n_{r}}{\underset{j=1}{\sum }}\frac{{\hat{\beta }}_{e,j}-\beta_{e}}{\beta_{e}}\times 100\%=\frac{{\bar{\beta }}_{e}-\beta_{e}}{\beta_{e}}\times 100\%,$$

where ${\hat{\beta }}_{e,j}$ is the estimate for β*_e_* at the *j*th simulation replicate, *n_r_* is the number of simulation replicates, and ${\bar{\beta }}_{e}=\frac{1}{n_{r}}\overset{n_{r}}{\underset{j=1}{\sum }}{\hat{\beta }}_{e,j}$. When the null hypothesis is true, i.e., β*_e_* = 0, the bias, ${\bar{\beta }}_{e}-\beta_{e}$, is reported instead. Using the same notations and letting SE stand for stand error, the 95% confidence interval for β*_e_* at the *j*th simulation replicate is (${\hat{\beta }}_{e,j}-z_{0.975}\times \text{SE}\left({\hat{\beta }}_{e,j}\right)$, ${\hat{\beta }}_{e,j}+z_{0.975}\times \text{SE}\left({\hat{\beta }}_{e,j}\right)$) and the corresponding width of 95% confidence interval is $2\times z_{0.975}\times \text{SE}\left({\hat{\beta }}_{e,j}\right)$. Over the simulation replicates, instead of taking average of widths of 95% confidence interval, we calculate the averaged width of 95% confidence interval by

(8) $$\text{Width of 95\% CI }=2\times z_{0.975}\times \text{SE}\left({\hat{\beta }}_{e}\right),$$

where $\text{SE}\left({\hat{\beta }}_{e}\right)=\sqrt{\frac{1}{n_{r}-1}\overset{n_{r}}{\underset{j=1}{\sum }}{\left({\hat{\beta }}_{e,j}-{\bar{\beta }}_{e}\right)}^{2}}$ is the estimated SE of ${\hat{\beta }}_{e}$, and *z*_0.975_ is the inverse cumulative density of the standard normal at 0.975. ${\hat{\beta }}_{e}$ is unbiased when the percent of bias is 0%. The width of 95% confidence interval is compared between models only when both percents of bias are within ±5%, which is considered acceptable.

The estimation results assuming the null hypothesis is true are presented in Tables 3 and 4. In Table 3, the bias is consistently around 0 regardless of confounding effect and age matching range. In Table 4, the width of 95% confidence interval does not vary significantly with age matching range and odds ratio associated with a 10-year increase in age. It remains similar between unconditional and conditional models until the mean age difference reaches 20 when the unconditional model has a shorter interval than the conditional model. The SE of ${\hat{\beta }}_{e}$ is around 0.15 across simulation settings under both models but reduces to 0.13 under the unconditional model when the mean age difference is 20. The reduction in the SE leads to the difference of 0.08 roughly in the width of 95% confidence interval.

**Table 3:** Biases of unconditional and conditional logistic regression models under the null hypothesis.

	Age distribution of unexposed and exposed subjects (in years)					
	*N*(65, 10^2^) vs. *N(*70, 10^2^)		*N*(60, 10^2^) vs. *N(*70, 10^2^)		*N*(50, 10^2^) vs. *N(*70, 10^2^)	
*d*	Unconditional	Conditional	Unconditional	Conditional	Unconditional	Conditional
**Odds ratio associated with a 10-year increase in age = 1**						
0	0.00	0.00	0.00	0.00	0.00	0.00
1	0.00	0.00	0.00	0.00	0.00	0.00
2	0.00	0.00	0.00	0.00	0.01	0.01
3	0.00	0.00	0.01	0.01	0.01	0.01
**Odds ratio associated with a 10-year increase in age = 1.5**						
0	0.00	0.00	0.00	0.00	0.00	0.00
1	0.00	0.00	0.00	0.00	0.00	0.00
2	0.00	0.00	0.00	0.00	0.00	0.00
3	0.00	0.00	0.01	0.01	0.00	0.00
**Odds ratio associated with a 10-year increase in age = 2**						
0	0.00	0.00	0.00	0.00	0.00	0.00
1	0.00	0.00	0.00	0.00	0.00	0.00
2	0.00	0.00	0.00	0.00	0.00	0.00
3	0.00	0.00	0.00	0.00	−0.01	0.00
**Odds ratio associated with a 10-year increase in age = 3**						
0	0.00	0.00	0.00	0.00	0.00	0.00
1	0.00	0.00	0.00	0.00	0.00	0.00
2	0.00	0.00	0.00	0.00	−0.01	−0.01
3	0.00	0.00	0.00	0.00	−0.02	−0.01

*Cases and controls were matched by age ± d. The odds ratio associated with the exposure was 1 under the null hypothesis, H_0_: β_e_ = 0. Numbers of matching sets were 400, 500, and 900 in the three scenarios of age distributions*.

**Table 4:** Widths of 95% confidence interval of unconditional and conditional logistic regression models under the null hypothesis.

	Age distribution of unexposed and exposed subjects (in years)					
	*N*(65, 10^2^) vs. *N(*70, 10^2^)		*N*(60, 10^2^) vs. *N(*70, 10^2^)		*N*(50, 10^2^) vs. *N(*70, 10^2^)	
*d*	Unconditional	Conditional	Unconditional	Conditional	Unconditional	Conditional
**Odds ratio associated with a 10-year increase in age = 1**						
0	0.60	0.61	0.57	0.58	0.52	0.60
1	0.58	0.59	0.60	0.61	0.51	0.59
2	0.62	0.63	0.58	0.60	0.50	0.58
3	0.61	0.62	0.59	0.60	0.50	0.58
**Odds ratio associated with a 10-year increase in age = 1.5**						
0	0.61	0.62	0.57	0.58	0.49	0.56
1	0.56	0.57	0.56	0.57	0.48	0.54
2	0.58	0.58	0.56	0.57	0.48	0.54
3	0.60	0.62	0.55	0.56	0.50	0.56
**Odds ratio associated with a 10-year increase in age = 2**						
0	0.57	0.58	0.56	0.57	0.50	0.56
1	0.59	0.61	0.53	0.54	0.50	0.57
2	0.57	0.59	0.56	0.57	0.51	0.58
3	0.60	0.61	0.56	0.58	0.52	0.59
**Odds ratio associated with a 10-year increase in age = 3**						
0	0.58	0.59	0.56	0.57	0.53	0.59
1	0.57	0.58	0.53	0.54	0.50	0.57
2	0.57	0.58	0.55	0.57	0.52	0.60
3	0.61	0.63	0.54	0.56	0.53	0.61

*Cases and controls were matched by age ± d. The odds ratio associated with the exposure was 1 under the null hypothesis, H_0_: β_e_ = 0. Numbers of matching sets were 400, 500, and 900 in the three scenarios of age distributions*.

The estimation results when the alternative hypothesis is true are presented in Table 5 (% of bias) and Table 6 (width of 95% confidence interval). When the mean age difference is 5, i.e., age distribution *N*(65, 10^2^) for unexposed subjects and *N*(70, 10^2^) for exposed subjects, both models consistently give similar percents of bias within the range of ±5% and also similar widths of 95% confidence interval. While the difference is negligible, the unconditional model consistently produces a shorter 95% confidence interval than the conditional model. The findings are consistent when the mean age difference is 10. When the mean age difference is 20, the unconditional model consistently underestimates β*_e_* with a percent of bias smaller than −5%, but the conditional model consistently produces a bias within ±5% range. The width of 95% confidence interval is not compared between models because the unconditional estimate is always biased.

**Table 5:** Percents of bias (%) of unconditional and conditional logistic regression models under the alternative hypothesis.

	Age distribution of unexposed and exposed subjects (in years)					
	*N*(65, 10^2^) vs. *N(*70, 10^2^)		*N*(60, 10^2^) vs. *N(*70, 10^2^)		*N*(50, 10^2^) vs. *N(*70, 10^2^)	
*d*	Unconditional	Conditional	Unconditional	Conditional	Unconditional	Conditional
**Odds ratio associated with a 10-year increase in age = 1**						
0	−1.36	−1.22	−0.40	0.95	**−10.95**	1.49
1	0.27	0.68	−1.53	−0.32	**−10.91**	1.61
2	−1.75	−1.33	−1.64	−0.11	**−10.79**	0.95
3	−1.04	−0.79	−0.23	1.38	**−9.07**	3.26
**Odds ratio associated with a 10-year increase in age = 1.5**						
0	0.14	0.17	0.31	0.91	**−7.62**	2.44
1	−3.93	−3.49	0.26	1.22	**−9.71**	0.15
2	2.48	3.17	0.28	1.43	**−8.39**	1.62
3	1.69	1.97	0.51	1.38	**−9.20**	0.89
**Odds ratio associated with a 10-year increase in age = 2**						
0	−0.10	−0.05	−1.85	−1.16	**−12.18**	−2.52
1	1.93	2.49	−1.82	−1.32	**−9.35**	0.78
2	0.06	0.66	−0.73	−0.20	**−10.24**	0.45
3	0.91	1.17	−0.26	0.22	**−13.36**	−3.13
**Odds ratio associated with a 10-year increase in age = 3**						
0	−2.16	−2.13	0.10	0.72	**−9.93**	0.30
1	0.84	1.38	0.34	1.22	**−10.94**	−0.08
2	0.08	0.39	−0.56	0.04	**−11.05**	0.99
3	1.77	1.98	1.15	1.67	**−15.28**	−2.68

*Cases and controls were matched by age ± d. The odds ratio associated with the exposure was 1.5 under the alternative hypothesis, H_0_: β_e_ ≠ 0. Numbers of matching sets were 400, 500, and 900 in the three scenarios of age distributions. Percents of bias out of ±5% range were highlighted in bold*.

**Table 6:** Widths of 95% confidence interval of unconditional and conditional logistic regression models under the alternative hypothesis.

	Age distribution of unexposed and exposed subjects (in years)					
	*N*(65, 10^2^) vs. *N(*70, 10^2^)		*N*(60, 10^2^) vs. *N(*70, 10^2^)		*N*(50, 10^2^) vs. *N(*70, 10^2^)	
*d*	Unconditional	Conditional	Unconditional	Conditional	Unconditional	Conditional
**Odds ratio associated with a 10-year increase in age = 1**						
0	0.60	0.61	0.57	0.58	0.52	0.60
1	0.58	0.59	0.60	0.61	0.51	0.59
2	0.62	0.63	0.58	0.60	0.50	0.58
3	0.61	0.62	0.59	0.60	0.50	0.58
**Odds ratio associated with a 10-year increase in age = 1.5**						
0	0.61	0.62	0.57	0.58	0.49	0.56
1	0.56	0.57	0.56	0.57	0.48	0.54
2	0.58	0.58	0.56	0.57	0.48	0.54
3	0.60	0.62	0.55	0.56	0.50	0.56
**Odds ratio associated with a 10-year increase in age = 2**						
0	0.57	0.58	0.56	0.57	0.50	0.56
1	0.59	0.61	0.53	0.54	0.50	0.57
2	0.57	0.59	0.56	0.57	0.51	0.58
3	0.60	0.61	0.56	0.58	0.52	0.59
**Odds ratio associated with a 10-year increase in age = 3**						
0	0.58	0.59	0.56	0.57	0.53	0.59
1	0.57	0.58	0.53	0.54	0.50	0.57
2	0.57	0.58	0.55	0.57	0.52	0.60
3	0.61	0.63	0.54	0.56	0.53	0.61

*Cases and controls were matched by age ± d. The odds ratio associated with the exposure was 1.5 under the alternative hypothesis, H_0_: β_e_ ≠ 0. Numbers of matching sets were 400, 500, and 900 in the three scenarios of age distributions*.

## Discussion

In conclusion, unconditional and conditional logistic regression models perform similarly in testing and estimation except when the age distributions of exposed and unexposed subjects are 20 years apart. When the two age distributions are 20 years apart, the unconditional model consistently gives a type I error below the acceptable range and is slightly less powerful than the conditional model under the alternative hypothesis. When the null hypothesis is true, the unconditional model unbiasedly estimates the effect of exposure and gives a shorter 95% confidence interval than the conditional model. When the alternative hypothesis is true, the unconditional model significantly underestimates the effect of exposure while the conditional model consistently produces an unbiased estimate.

When the mean age of exposed subjects is 20 years older than that of unexposed subjects, cases are more likely to be matched to controls with the same exposure status and the association is diminished accordingly. The unconditional method ignores matching but adjusts for confounding in the framework of regression. In general, the Mantel–Haenszel estimator and the logit-based estimator are similar when the data within strata, here age groups, are not too sparse (11). Without losing generalizability, assume that age is grouped into a few age groups. The data of each age group can be organized in a 2 by 2 table of exposure status (exposed/unexposed) vs. disease status (case/control) (see Table 7).

**Table 7:** 2 by 2 table of exposure status vs. disease status.

	Case	Control
Exposed	*a*	*b*
Unexposed	*c*	*d*

Denoted by *a, b, c*, and *d*, the four cell counts representing the numbers of exposed cases, exposed controls, unexposed cases, and unexposed controls, respectively. The Mantel–Haenszel odds ratio is given by

(9) $${\hat{\psi }}_{\text{MH}}=\underset{i}{\sum }\frac{a_{i}d_{i}}{n_{i}}/\frac{b_{i}c_{i}}{n_{i}},$$

where *i* is the index of age group. The top and bottom age groups particularly have the ratio of number of cases to number of controls given the exposure status close to the case–control matching ratio. The addition from a particular age group to the numerator and the denominator tend to be similar, which drives the association toward the null value.

In simulations, we fixed the disease prevalence at 10% and the exposure frequency at 30%. However, we do not expect that the relative performance of unconditional and conditional logistic regression models will change with varying disease prevalence and/or exposure frequency. The sample size (the number of matching sets) needed to achieve 80% power at the 5% significance level depends on the disease prevalence and exposure frequency. Through simulations, we assumed well-powered studies, and every case can be matched to a control, which is reasonable because the question that we attempt to address is whether a matched case–control data need to be analyzed by conditional logistic regression model. When the disease prevalence or the exposure frequency is lower, a larger sample size is needed to maintain 80% power, but the sample size for both methods is the same. The sample size is chosen for a combination of disease prevalence and exposure frequency to ensure 80% power. For a sufficiently large sample size regardless of disease prevalence and exposure frequency, our conclusions are generalizable for other disease prevalence and exposure frequency. Again, the objective of this article is to compare the two methods given a matched case–control data instead of unmatched and matched data from different study designs where matched data tend to have a smaller sample size due to unmatched cases.

Our findings suggest that when cases and controls are matched on age only, the data are essentially loose-matching data, and unconditional logistic regression is a proper method when the age distributions of exposed and unexposed subjects are not significantly apart. Previous literature has provided in-depth discussion about the advantages of unconditional regression model compared to its conditional alternative, such as convenience, easy to access, straightforward interpretation, and the potential to preserve unmatched controls (12). We argue that matched case–control studies have been underappreciated by the misconception that matched case–control data can be analyzed only by matched methods. A paper reviewed statistical methods of 37 matched case–control studies published in 2010. Among these studies, a majority of them performed matching on demographic variables namely age and sex only. It was concluded that less than half studies (43%) were analyzed with proper statistical techniques (14). The conclusion was made as the authors claimed following the book of Breslow et al. (1), where a Mantel–Haenszel matched-pairs analysis or conditional logistic regression was expected for dichotomous outcomes. Based on our findings, matched methods are not necessary for loose-matching data, e.g., data matched on a small number of demographic variables. While we believe that it is realistically rare to observe two age distributions that are 20 years apart for exposed and unexposed subjects, it gives us an example how the matching distortion (matched cases and controls tend to share the same exposure status) fails the unconditional logistic regression model. In contrast, the matching distortion was corrected by including the matching variables in the conditional logistic regression model (12, 13). Although we only considered a single matching variable, i.e., age, our findings can be generalized for matching on sex and age that apparently produces loose-matching data. With an increasing number of matching variables, loose matching is less likely to hold in the data, e.g., matching variables used in the study by Jenab et al. (15): age, gender, study center, time of day at blood collection, and duration of fasting at blood collection; women were further matched by menopausal status, phase of menstrual cycle at the time of blood collection, and use of hormone replacement therapy. However, the strength of loose matching is not always reflected from the number of matching variables. Matching on neighborhood or matching based on relationships implicitly matches numerous unmeasured variables including unmeasurable variables. Such studies apparently generate genuinely matched data that need to be analyzed by matched methods. It should be cautioned that our findings are for matched case–control data and cannot be generalized for propensity score (PS) matched data. PS method was developed to facilitate causal inference in the spirit of clinical trials (16). Matching in PS method is performed on the probability of a treatment assignment, which is determined by a selection of variables including confounders. After controlling for these variables, it is assumed that the outcome is independent of treatment status. The study is typically a cohort study, and the purpose of PS matching is to ensure that the treatment groups are balanced with respect to the variables (conditional independence). In contrast, case–control studies are retrospective studies, and the exposure status is observed. While there is a debate about whether treated and untreated samples should be regarded as independent, which will inform the choice of statistical methods (17), it is different from the question that we have tried to address in terms of study design and matching scheme.

The scope of this study is limited to case–control studies that perform matching on a few demographic variables and consider methods of unconditional and conditional logistic regression models. In addition, the simulation settings assume absolute matching success, no model misspecification, and no interaction between exposure and matching variables. However, these assumptions can be relaxed and will require further investigation. An unpublished data of Kuo’s collaborator was collected to assess placental telomere length in preterm fetal growth restriction where each preterm fetal growth restriction case was matched to two controls by gestational age within 6 days of deliveries. The results by a linear regression model (unmatched method) and a linear mixed effects model assuming random effects for matching sets (matched method) were quite similar in terms of regression coefficient and *P* value associated with the case–control status, which supports our finding that case–control data matched on a few demographic variables can be properly analyzed by unmatched methods. To conclude, it has been known that matched methods, e.g., conditional logistic regression, are required for genuinely matched case–control data to tackle the sparse data problem. Matched methods additionally are robust to the matching distortion. Unmatched methods, e.g., unconditional logistic regression, are viable options for loose-matching data based on our findings. When the study design involves other complex features such as censoring and repeated measures, matching on a few demographic variables can be ignored if the confounding effect is not very large. Standard methods such as Cox regression and generalized estimating equation then can be readily applied. Unmatched methods also are appealing for saving computational time when the same analysis needs to be repeated extensively, e.g., genome-wide association analysis. In addition to matching, other factors also need to be considered, such as study design and practical feasibility when choosing a statistical method.

## Author Contributions

All of the authors contributed significantly to study design, result interpretation, and manuscript preparation. The data simulations were conducted by C-LK.

## Conflict of Interest Statement

The authors declare that the research was conducted in the absence of any commercial or financial relationships that could be construed as a potential conflict of interest.

## References

[1] Breslow NE Day NE Halvorsen KT Prentice RL Sabai C. Estimation of multiple relative risk functions in matched case-control studies. Am J Epidemiol (1978) 108(4):299–307. 10.1093/oxfordjournals.aje.a112623 727199

[2] Costanza MC. Matching. Prev Med (1995) 24(5):425–33. 10.1006/pmed.1995.1069 8524715

[3] Kupper LL Karon JM Kleinbaum DG Morgenstern H Lewis DK. Matching in epidemiologic studies: validity and efficiency considerations. Biometrics (1981) 37(2):271–91. 10.2307/2530417 7272415

[4] Miettinen OS Estimation of relative risk from individually matched series. Biometrics (1970) 26(1):75–86. 10.2307/2529046 5461791

[5] Wacholder S Silverman DT McLaughlin JK Mandel JS. Selection of controls in case-control studies. II. Types of controls. Am J Epidemiol (1992) 135(9):1029–41. 10.1093/oxfordjournals.aje.a116397 1595689

[6] McKinlay SM. Pair-matching—a reappraisal of a popular technique. Biometrics (1977) 33(4):725–35. 10.2307/2529471 588658

[7] Thomas DC Greenland S. The relative efficiencies of matched and independent sample designs for case-control studies. J Chronic Dis (1983) 36(10):685–97. 10.1016/0021-9681(83)90162-5 6630405

[8] Thompson WD Kelsey JL Walter SD. Cost and efficiency in the choice of matched and unmatched case-control study designs. Am J Epidemiol (1982) 116(5):840–51. 10.1093/oxfordjournals.aje.a113475 7148807

[9] Woodward M Epidemiology: Study Design and Data Analysis. Boca Raton: Chapman & Hall/CRC (2005). 849 p.

[10] Greenland S Schwartzbaum JA Finkle WD. Problems due to small samples and sparse data in conditional logistic regression analysis. Am J Epidemiol (2000) 151(5):531–9. 10.1093/oxfordjournals.aje.a010240 10707923

[11] Hosmer DW Lemeshow S Sturdivant RX Applied Logistic Regression. 3rd ed (Vol. 398). Hoboken, NJ: Wiley (2013).

[12] Pearce N Analysis of matched case-control studies. BMJ (2016) 352:i969 10.1136/bmj.i969 26916049 PMC4770817

[13] Rothman KJ Greenland S Lash TL Modern Epidemiology, Thoroughly Rev and Updated. Philadelphia: Wolters Kluwer Health/Lippincott Williams & Wilkins (2008). 758 p. Available from: http://www.loc.gov/catdir/enhancements/fy0743/2007036316-d.html; http://www.loc.gov/catdir/enhancements/fy0828/2007036316-t.html

[14] Niven DJ Berthiaume LR Fick GH Laupland KB. Matched case-control studies: a review of reported statistical methodology. Clin Epidemiol (2012) 4:99–110. 10.2147/CLEP.S30816 22570570 PMC3346204

[15] Jenab M Bueno-de-Mesquita HB Ferrari P van Duijnhoven FJ Norat T Pischon T Association between pre-diagnostic circulating vitamin D concentration and risk of colorectal cancer in European populations: a nested case-control study. BMJ (2010) 340:b5500. 10.1136/bmj.b5500 20093284 PMC2809840

[16] Austin PC. An introduction to propensity score methods for reducing the effects of confounding in observational studies. Multivariate Behav Res (2011) 46(3):399–424. 10.1080/00273171.2011.568786 21818162 PMC3144483

[17] Stuart EA Ialongo NS. Matching methods for selection of subjects for follow-up. Multivariate Behav Res (2010) 45(4):746–65. 10.1080/00273171.2010.503544 21221424 PMC3017384

